# Radiomic features and carotid stenosis in periodontitis a two stage bootstrap and multimodal machine learning study

**DOI:** 10.1038/s41598-026-38463-1

**Published:** 2026-02-10

**Authors:** Mengqiang Zhang, Jing Cai, Qian Cao, Zhipeng Chen, Subinuer Maimaitiaili, Shaoxun Yuan, Tao Yang, Zhen Li, Zhen Zhang, Yun Yang, Tong Qiao

**Affiliations:** 1 Department of Vascular Surgery, Nanjing Drum Tower Hospital, Affiliated Hospital of Medical School, Nanjing University, Nanjing, Jiangsu China; 2 School of Public Health/Collaborative Innovation Center for Cancer Personalized Medicine, Nanjing Medical University, Nanjing, Jiangsu China; 3 Zhejiang Cancer Hospital, Hangzhou Institute of Medicine (HIM), Chinese Academy of Sciences, Hangzhou, Zhejiang China; 4 School of Artificial Intelligence and Information Technology, Nanjing University of Chinese Medicine, Nanjing, Jiangsu China; 5 Department of Implantology, Nanjing Stomatological Hospital, Medical School of Nanjing University, Nanjing, Jiangsu China; 6 Department of Stomatology, Nanjing Drum Tower Hospital, Affiliated Hospital of Medical School, Nanjing University, Nanjing, Jiangsu China

**Keywords:** Radiomics, Carotid atherosclerosis, Periodontitis, Machine learning, Early diagnosis, Biomarkers, Cardiology, Computational biology and bioinformatics, Diseases, Medical research, Risk factors

## Abstract

This study aims to develop and validate a deep learning model based on Cone Beam Computed Tomography (CBCT) radiomic features to achieve early detection of potential carotid atherosclerosis in periodontitis patients. The study utilised data from 279 observations, each with 206 features, to distinguish between periodontitis patients with and without concomitant carotid atherosclerosis. To address class imbalance, Synthetic Minority Over-sampling Technique(SMOTE) oversampling was applied (dup_size = 1), increasing the sample size to 390 observations. A bootstrap method (n_bootstrap = 1000) was employed for feature selection. In each iteration, a dataset was created by resampling with replacement. Features were first filtered using Spearman’s rank correlation to remove redundant variables (correlation coefficient > 0.8), followed by Lasso regression with ten-fold cross-validation to select predictive variables based on non-zero coefficients. High-frequency features identified through 1000 iterations underwent a second round of bootstrap analysis, where Logistic Regression combined with the Akaike Information Criterion (AIC) was used to determine the final variable set. This rigorous process ensured optimal feature selection for developing an effective early detection model for carotid atherosclerosis in periodontitis patients. The study analyzed data from 279 observations, with each observation characterized by 206 features, to differentiate between periodontitis patients with concurrent carotid atherosclerosis and those without. After SMOTE oversampling, the dataset was increased to 390 observations. As stated in the Methods, SMOTE was applied after baseline analysis to augment the dataset for model development. Feature selection through bootstrap methods identified 26 high-frequency features (> 500 times), which were further refined to a final set of 20 features using Logistic Regression combined with AIC. Three machine learning models—Logistic Regression (LR), Support Vector Machine (SVM), and Random Forest (RF)—were developed and evaluated using five-fold cross-validation. The best-performing model was the RF model, achieving an Area Under the Curve(AUC) of 0.892, sensitivity of 0.957, specificity of 0.710, and accuracy of 0.859. Receiver Operating Characteristic(ROC) curves and calibration plots demonstrated good predictive performance and model calibration across all three models. Decision curve analysis showed that the RF model provided the highest net benefit across a range of risk thresholds, indicating its potential for clinical utility in early detection of carotid atherosclerosis in periodontitis patients. This study developed a random forest model using CBCT radiomics to detect carotid atherosclerosis in periodontitis patients early. After rigorous feature selection and five-fold cross-validation, it achieved an AUC of 0.892, with sensitivity of 0.957 and specificity of 0.710. The model shows high predictive performance and clinical utility, offering an effective tool for early detection.

## Introduction

In the dynamic field of cardiovascular disease (CVD) management, early detection remains pivotal for reducing morbidity and mortality^1–3^. Among various manifestations of atherosclerosis, carotid artery stenosis is a critical risk factor for ischemic stroke, posing significant challenges to global healthcare systems^4,5^. Emerging evidence highlights a crucial link between periodontitis—an inflammatory condition affecting tooth-supporting structures and an increased risk of cardiovascular events, including carotid atherosclerosis^6–12^. Moreover, in mice with atherosclerosis, periodontal inflammation was more severe, and the unique changes in lipid profiles observed in these mice were closely related to the aforementioned findings^13^. Despite these compelling associations, there is still a substantial unmet need for effective early detection methods tailored to this high-risk population.

Cone Beam Computed Tomography (CBCT), traditionally used in dental imaging, is increasingly recognized for its potential to provide detailed anatomical insights relevant to oral health^14,15^. The rapidly advancing field of radiomics-characterized by the extraction of extensive quantitative features from medical images-promises unprecedented precision in disease characterization, potentially revolutionizing diagnostic accuracy and patient outcomes. However, the application of CBCT radiomic features in predictive models for detecting carotid atherosclerosis among periodontitis patients remains largely unexplored.

This study aims to pioneer the development and validation of a deep learning model leveraging CBCT radiomic features to detect early signs of carotid atherosclerosis in patients with periodontitis. Our methodology integrates state-of-the-art machine learning algorithms, including Random Forest, Support Vector Machines, and Logistic Regression, with sophisticated feature selection techniques such as SMOTE oversampling, Spearman’s rank correlation, Lasso regression, and Akaike Information Criterion (AIC). This rigorous approach not only enhances the identification of individuals at heightened risk but also deepens our understanding of the complex interplay between periodontal and systemic vascular health.

## Materials and methods

### Datasets

This retrospective study was approved by the Ethics Committee of Nanjing Drum Tower Hospital (approval number: 2023-461-02), and the requirement for written informed consent was waived. In this study, a total of 279 patients were included for analysis, comprising 168 cases of carotid artery stenosis with periodontitis and 111 cases of periodontitis alone. To address the issue of data imbalance, we applied the Synthetic Minority Over-sampling Technique (SMOTE) with a duplication size parameter set to 1 (dup_size = 1). After resampling, the number of periodontitis-only cases increased to 222, resulting in a total sample size of 390 cases. It is important to note that all baseline characteristic analyses (Table 1) were performed on the original, non-augmented dataset (*n* = 279) to accurately reflect the real-world patient population. The SMOTE procedure was applied exclusively to the feature dataset for the purpose of training and validating the machine learning models, after the baseline comparison was completed. The study was conducted in accordance with the principles set forth in the Declaration of Helsinki.

### CBCT-Based diagnosis of periodontitis

The diagnosis of periodontitis was established radiographically using CBCT scans, following the consensus criteria of the 2018 Classification of Periodontal Diseases^16^. The evaluation was independently performed by two blinded examiners (with inter-examiner reliability reported as Kappa = 0.75).

Periodontitis was defined by the presence of interdental bone loss (IBL) affecting at least two non-adjacent teeth. Bone loss was quantified by measuring the distance from the Cementoenamel Junction (CEJ) to the Alveolar Bone Crest (ABC) on multiplanar reconstructed images. A site was considered positive for periodontitis-associated bone loss if the CEJ-ABC distance was ≥ 3 mm at its deepest point, in the absence of other clear causes (e.g., periapical pathology). Subjects meeting this criterion in at least two quadrants were included in the periodontitis group. This binary classification (presence/absence of periodontitis) formed the basis for the subsequent association analysis with carotid stenosis.

### Inclusion and exclusion criteria

#### Inclusion criteria

Patients were included if they met all of the following criteria:

- Diagnosed with carotid artery stenosis by carotid duplex ultrasound or computed tomography angiography (CTA);
- Aged between 18 and 85 years (inclusive);
- Underwent a contemporaneous jawbone cone-beam computed tomography (CBCT) scan.

#### Exclusion criteria

Patients were excluded based on any of the following criteria:

- Presence of severe artifacts or poor-quality jawbone CBCT images that would compromise accurate segmentation of the region of interest (ROI);
- History of jawbone tumors, cysts, osteomyelitis, osteoradionecrosis, or major jaw surgery;
- Acute orofacial infection or surgery in the relevant area within the 3 months prior to the study;
- Unavailability of key clinical data or data required for assessing plaque stability;
- Diagnosis of a systemic disease known to severely affect bone metabolism (e.g., hyperparathyroidism).

### Grouping method

The diagnosis of periodontitis was confirmed by oral medicine specialists using CBCT, while carotid artery stenosis was diagnosed by vascular surgeons based on carotid ultrasound or carotid artery angiography. Patients with both periodontitis and carotid atherosclerosis were grouped together, and those with periodontitis alone formed a separate group.

### CBCT image acquisition

For CBCT imaging, we used the NewTom VGI Tomograph (NewTom, Verona, Italy), which references the Frankfurt plane and supports 360-degree rotation. The system automatically adjusts the X-ray dose based on the patient’s anatomy to optimise image quality while minimising radiation exposure. Imaging parameters were: 110 kVp, 40–50 mA; high-resolution mode with a 0.3 mm focal spot; exposure time of 5.4 s; and slice thickness of 0.25 mm, ensuring detailed visualisation of anatomical structures.

### Whole periodontal region segmentation

**Fig. 1:**
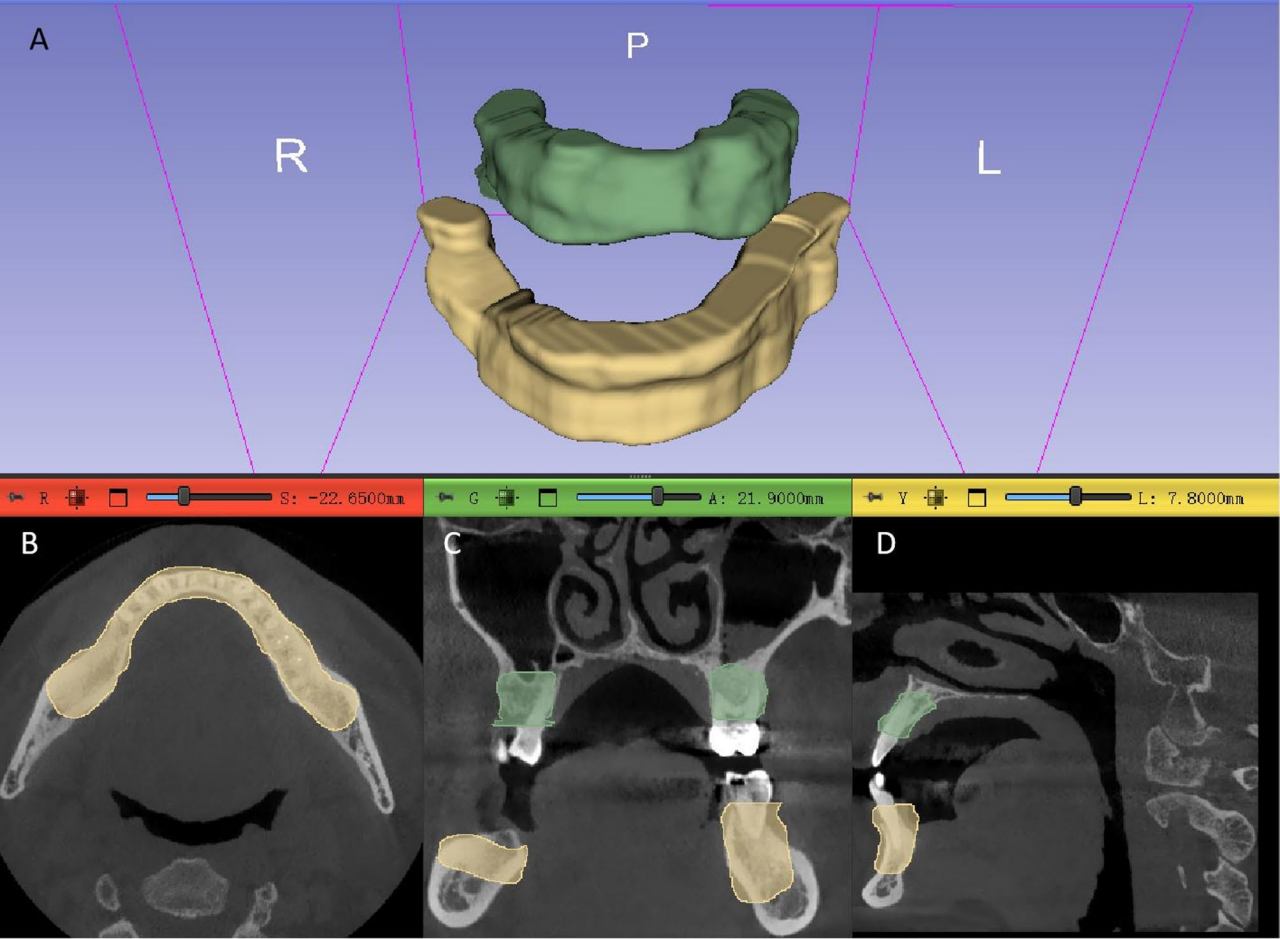
A schematic illustration from a Cone Beam Computed Tomography (CBCT) scan of a patient, highlighting the outlines of the periodontal tissues. **A**: 3D view of the regions of interest. **B**: Axial view of the CBCT. **C**: Coronal view of the CBCT. **D**: Sagittal view of the CBCT.

The mandibular and maxillary regions were segmented and subsequently merged into a single region of interest (ROI). This process was performed using 3D Slicer software(www.slicer.org, version 5.6.2) by an oral and maxillofacial specialist with 10 years of experience. Region of Interest (ROI) Definition for Periodontal Analysis: Maxilla: The upper boundary is defined by the line connecting the lower wall of the maxillary sinus and the floor of the nose to the lower wall of the contralateral maxillary sinus. The lower boundary is the line connecting all the enamel-cementum junctions of the teeth. Mandible: The upper boundary is the line connecting all the enamel-cementum junctions of the teeth. The lower boundary is defined by the line connecting the inferior alveolar canals bilaterally in the posterior region and the bilateral mental foramen in the anterior region. Multiplanar Reconstruction (MPR) Views (Axial, Sagittal, Coronal) for Both Maxilla and Mandible: The boundaries include the labial and buccal alveolar bone plates and the lingual or palatal bone plates(Fig. 1). The segmentation results were independently reviewed and any inaccuracies were corrected to ensure the highest quality of the delineated anatomical regions.

### Feature selection

The Pyradiomics module in Python(3.7.16) was used to extract features from the regions of interest (ROIs), with a total of 206 features extracted for each patient. To identify the most relevant features for our analysis, we employed a robust feature selection procedure based on the bootstrap method. The process is described in detail below:

### Bootstrap sampling and initial feature screening

We performed bootstrap sampling with n_bootstrap = 1000. Each bootstrap sample dataset was generated by randomly sampling with replacement from the original dataset, where the number of samples equaled the number of rows in the original dataset (nrow(data)). Within each bootstrap iteration, the following operations were conducted:

#### Spearman rank correlation analysis

To eliminate redundancy among features, we calculated the Spearman rank correlation coefficients between all pairs of variables. Variables with a correlation coefficient greater than 0.8 were considered redundant, and the variable with the weaker correlation to the outcome variable was removed.

### Lasso regression

Lasso (Least Absolute Shrinkage and Selection Operator) regression was applied to further reduce the dimensionality of the predictor variables and select the most relevant features. To determine the optimal number of variables and avoid overfitting, we performed ten-fold cross-validation to select the optimal regularization parameter λ. Variables with non-zero coefficients in the final Lasso model were retained.

The above process was repeated 1000 times, and the frequency of each feature being selected across all bootstrap iterations was recorded. Features that appeared with higher frequency were carried forward to the second stage of the bootstrap process.

### Secondary bootstrap process with logistic regression and AIC

In the second stage, we performed another round of bootstrap sampling (n_bootstrap = 1000) to refine the feature selection:

#### Logistic regression with AIC-Based variable selection

Using the features with higher selection frequencies from the first stage, we constructed multivariate logistic regression models. A stepwise regression approach was applied to identify the optimal combination of variables, guided by the Akaike Information Criterion (AIC). The frequency of each variable combination appearing across all bootstrap iterations was recorded.

This process was repeated 1000 times, and the variable combination with the highest selection frequency was chosen for subsequent modeling.

### Statistical analysis

Statistical analysis was conducted utilizing SPSS software(version 27.0) and Python software (version 3.7.16). For comparisons between two groups, continuous variables that adhered to a normal distribution were reported as mean ± standard deviation (‾x ± s) and analyzed using independent samples t-tests. Conversely, continuous variables that did not conform to a normal distribution were presented as median (interquartile range) [M(IQR)] and analyzed using the Mann-Whitney U test. Categorical variables were expressed as counts or percentages and analyzed using the chi-squared (χ²) test.

## Results

A total of 279 patients were included in the study, of which 168 patients had carotid stenosis combined with periodontitis, and 111 patients had periodontitis alone. The baseline characteristics of the two groups are presented in Table 1. Among the baseline characteristics, a history of previous stroke showed a statistically significant difference between the two groups (*P* = 0.04), while no significant differences were observed in other variables.

**Table 1:** Baseline characteristics of 279 patients with carotid stenosis and Periodontitis.

Age-y	CAS with periodontitis group(*n* = 168)	Periodontitis group(*n* = 111)	*P* value
	65.09 ± 9.81	66.69 ± 8.68	0.62
Male sex	90(53.6%)	72(64.9%)	0.31
BMI– kg/m^2^	21.03(20.28, 25.10)	21.97(20.14, 24.82)	0.32
Smoking	71(42.2%)	52(46.8%)	0.68
Stroke	85(50.6%)	48(43.2%)	0.04
Hypertension	95(56.5%)	55(49.5%)	0.28
Diabetes	68(40.5%)	42(37.8%)	0.25
Hypercholesterolaemia	20(11.9%)	17(10.1%)	0.21
Coronary disease	43(25.6%)	36(32.3%)	0.68
Creatinine-umol/L	63(55,74)	62(55,74)	0.87
Interleukin-6-pg/ml	4.87(4.32,5.6)	4.77(4.32,9.23)	0.38
CRP-mg/L	3.8(2.1,7.5)	3.7(2.4,6.7)	0.74
ESR-mm/h	16(12,18)	15(6,18)	0.08
BNP-pg/ml	27(18.9,36.6)	28(18.9,55.2)	0.78
Homocysteine-umol/L	12.9(10.7,15.3)	14.15(11,15.3)	0.23
LDL Cholesterol-mmol/L	1.16(1.04,1.38)	1.16(1.00,1.36)	0.48
Triglycerides-mmol/L	1.07(0.88,1.37)	1.08(0.75,1.51)	0.77

CAS=Carotid artery stenosis. BMI=Body mass index. LDL = low-density lipoprotein. ESR=Erythrocyte sedimentation rate. CRP = C-reactive protein. BNP = B-type Natriuretic Peptide.

### Feature selection and stability analysis

A rigorous two-stage bootstrap approach (*n* = 1000 iterations) was employed to identify robust radiomic features predictive of concomitant carotid artery stenosis in periodontitis patients.

### First-stage feature reduction

Spearman rank correlation (*p* > 0.8 threshold) and LASSO regression with 10-fold cross-validation were applied to eliminate redundant variables. This process yielded 26 high-frequency features (selection count > 500), predominantly comprising: Morphometric parameters: Original_shape_Sphericity_1/2, Original_shape_Elongation_1/2, and minimum axis lengths. First-order intensity statistics: Original_firstorder_Kurtosis_1, median values, and interquartile ranges. Higher-order texture features: Gray-level co-occurrence matrix (GLCM) metrics (DifferenceVariance, MaximumProbability), Gray-level size zone matrix (GLSZM) large-area emphasis descriptors, Neighborhood gray-tone difference matrix (NGTDM) contrast and strength.

Notably, bilateral feature symmetry was observed, with morphometric and textural markers concurrently appearing in both maxillary and mandibular regions (Table 2), suggesting systemic pathophysiological influences.

**Table 2:** High-frequency radiomic features (selection count > 500) from initial bootstrap screening. Blue highlighting indicates bilateral manifestations.

Feature	Frequency
**Original_shape_Elongation_2**	997
**Original_shape_Sphericity_1**	918
Original_ngtdm_Contrast_2	918
Original_shape_LeastAxisLength_2	890
**Original_shape_Sphericity_2**	878
Original_firstorder_Kurtosis_1	828
**Original_shape_Elongation_1**	798
Original_firstorder_Median_1	794
Original_glszm_LargeAreaLowGrayLevelEmphasis_2	792
Original_glcm_DifferenceVariance_1	789
Original_glcm_MaximumProbability_1	787
Original_firstorder_InterquartileRange_2	763
**Original_shape_Maximum2DDiameterRow_1**	724
**Original_shape_Maximum2DDiameterRow_2**	711
**Original_shape_MinorAxisLength_1**	710
Original_firstorder_RobustMeanAbsoluteDeviation_1	703
Original_shape_Maximum2DDiameterSlice_2	701
Original_shape_Flatness_1	691
Original_firstorder_90Percentile_2	659
**Original_firstorder_Skewness_1**	599
Original_glcm_Imc2_1	585
Original_glszm_LargeAreaHighGrayLevelEmphasis_2	563
**Original_shape_MinorAxisLength_2**	550
**Original_firstorder_Skewness_2**	525
Original_glcm_Correlation_2	521
Original_ngtdm_Strength_2	505

The features in bold appear with high frequency in both the upper and lower .

Second-stage optimal feature selection.

A multivariate logistic regression model with AIC-based stepwise refinement was iterated (*n* = 1000 bootstraps), converging on a 20-feature signature with maximal predictive stability (Table 3). The final ensemble retained 85% of first-stage features, confirming their discriminant validity, while introducing additional texture biomarkers.

**Table 3:** Consensus feature combination from second-stage bootstrap analysis, ranked by selection frequency.

Feature	Frequency
original_firstorder_90Percentile_2	5
original_firstorder_InterquartileRange_2	
original_firstorder_Kurtosis_1	
original_firstorder_Median_1	
original_firstorder_RobustMeanAbsoluteDeviation_1	
original_firstorder_Skewness_1	
original_glcm_Correlation_2	
original_glcm_DifferenceVariance_1	
original_glcm_Imc2_1	
original_glszm_LargeAreaHighGrayLevelEmphasis_2	
original_ngtdm_Contrast_2	
original_ngtdm_Strength_2	
**original_shape_Elongation_1**	
**original_shape_Elongation_2**	
original_shape_Flatness_1	
original_shape_LeastAxisLength_2	
original_shape_Maximum2DDiameterSlice_2	
original_shape_MinorAxisLength_2	
**original_shape_Sphericity_1**	
**original_shape_Sphericity_2**	

The features in bold appear with high frequency in both the upper and lower .

### Predictive model performance

Three machine learning architectures were benchmarked via stratified 5-fold cross-validation: Cross-validation performance metrics.

### Cross-validation performance

In this study, we evaluated the performance of three machine learning models—Logistic Regression (LR), Support Vector Machine (SVM), and Random Forest (RF)—using various performance metrics across different folds. The results are summarized in Table 4. The Area Under the Curve (AUC), sensitivity, specificity, and accuracy for each model are presented for each fold. Notably: For Logistic Regression (LR), the highest AUC was observed in Fold 2 with an AUC of 0.874. For Support Vector Machine (SVM), the highest AUC was observed in Fold 3 with an AUC of 0.897. For Random Forest (RF), the highest AUC was observed in Fold 3 with an AUC of 0.891.

**Fig. 2:**
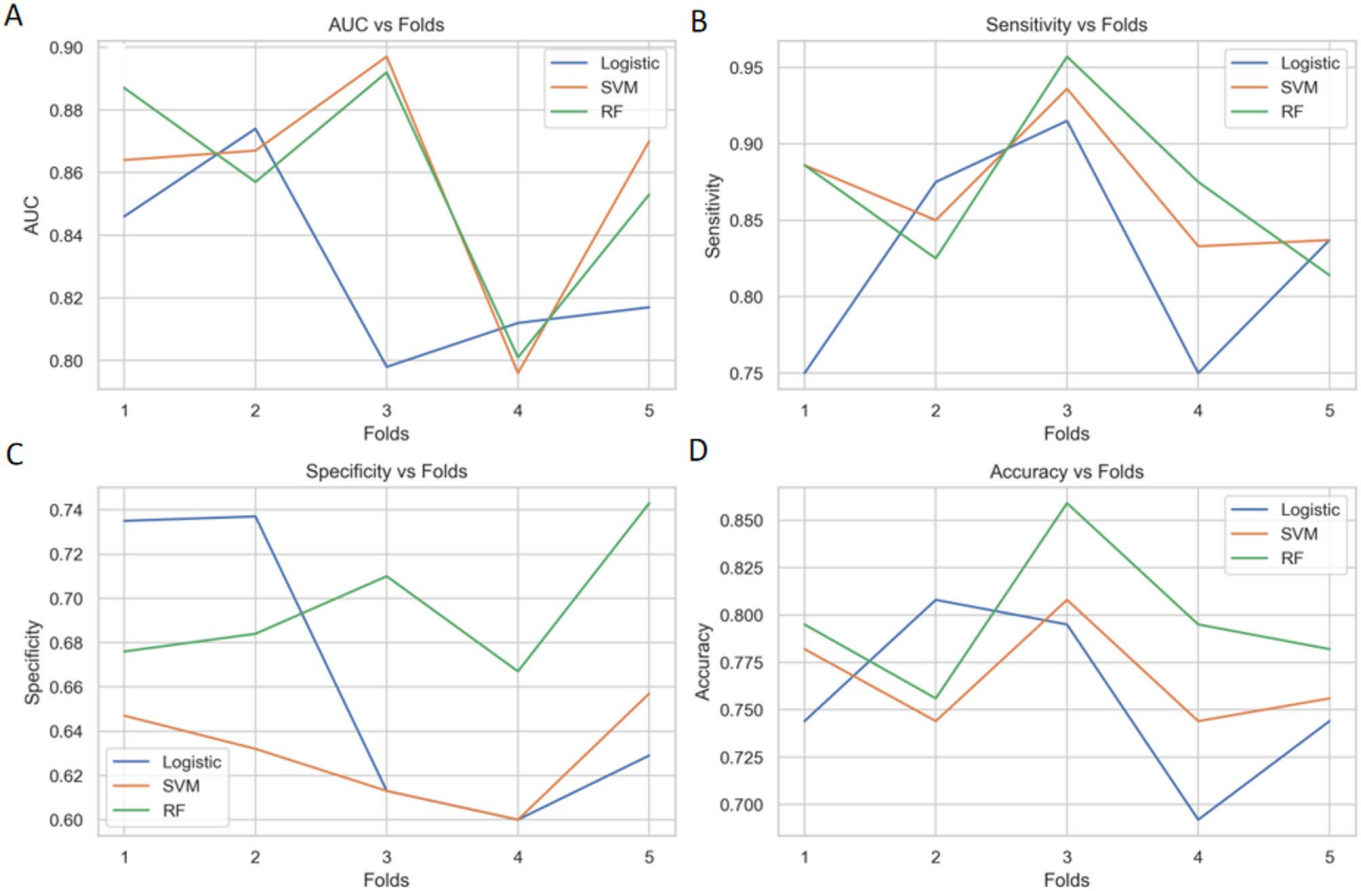
Comprehensive Evaluation of Machine Learning Model Performance: A Comparative Analysis Based on AUC, Sensitivity, Specificity, and Accuracy. (A) Comparison of AUC across different cross-validations. (B) Comparison of sensitivity across different cross-validations. (C) Comparison of specificity across different cross-validations. (D) Comparison of accuracy across different cross-validations.

Overall, the Random Forest model consistently showed higher AUC values compared to LR and SVM across most folds, indicating its superior performance in distinguishing between positive and negative cases. The RF model also demonstrated high sensitivity and specificity, particularly in Fold 3, where it achieved an AUC of 0.891, a sensitivity of 0.957, and a specificity of 0.710(Fig. 2).

**Table 4:** Performance metrics of three machine learning models across different Folds.

Method	Folds	AUC	Sensitivity	Specificity	Accuracy
Logistic	1	0.846	0.750	0.735	0.744
SVM	1	0.864	0.886	0.647	0.782
RF	1	0.887	0.886	0.676	0.795
Logistic	**2**	**0.874**	0.875	0.737	0.808
SVM	2	0.867	0.850	0.632	0.744
RF	2	0.857	0.825	0.684	0.756
Logistic	3	0.798	0.915	0.613	0.795
SVM	**3**	**0.897**	0.936	0.613	0.808
RF	**3**	**0.892**	0.957	0.710	0.859
Logistic	4	0.812	0.750	0.600	0.692
SVM	4	0.796	0.833	0.600	0.744
RF	4	0.801	0.875	0.667	0.795
Logistic	5	0.817	0.837	0.629	0.744
SVM	5	0.870	0.837	0.657	0.756
RF	5	0.853	0.814	0.743	0.782

SVM = Support Vector Machine. RF = Random Forest.

**Fig. 3:**
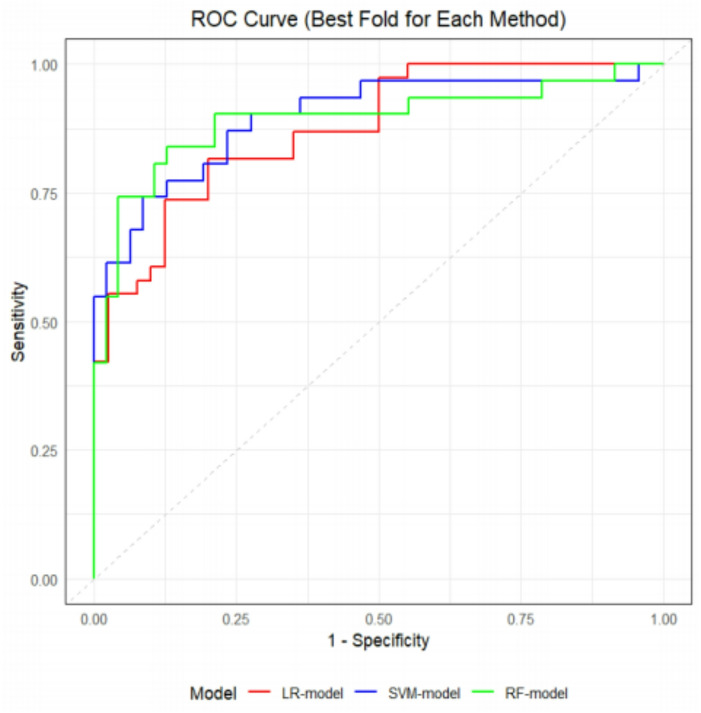
ROC and Calibration Curves for Three Machine Learning Models. (A) ROC Curves: The Receiver Operating Characteristic (ROC) curves evaluate the diagnostic accuracy of three machine learning models: Logistic Regression (LR), Support Vector Machine (SVM), and Random Forest (RF). The sensitivity is plotted against 1 - specificity. The LR-model, SVM-model, and RF-model are represented by red, blue, and green lines, respectively. The dashed line represents chance performance (AUC = 0.5). (B) Calibration Curves: The calibration curves assess the agreement between predicted probabilities and observed frequencies for the same models. The observed event percentage is plotted against the predicted probability (midpoint of bin). The LR-model, SVM-model, and RF-model are represented by red, blue, and green lines, respectively. Perfect calibration follows the diagonal.

In this study, we developed three machine learning models—Logistic Regression (LR), Support Vector Machine (SVM), and Random Forest (RF)—using selected features. Their diagnostic accuracy and calibration were evaluated using ROC curves and calibration plots, respectively. Figure 3A shows the ROC curves, plotting sensitivity against 1 - specificity for the LR-model (red), SVM-model (blue), and RF-model (green). The dashed line represents chance performance (AUC = 0.5). The RF-model demonstrates the highest AUC, indicating superior diagnostic accuracy across all risk thresholds, with notable advantages at both lower and higher thresholds. Figure 3B displays calibration curves, comparing predicted probabilities to observed event frequencies. The RF-model aligns closest to the diagonal, reflecting excellent calibration, while the LR-model and SVM-model show reasonable but less precise calibration. These results highlight the Random Forest model’s potential as a robust tool for clinical decision-making, excelling in both diagnostic accuracy and probability calibration.

### Clinical decision impact

**Fig. 4:**
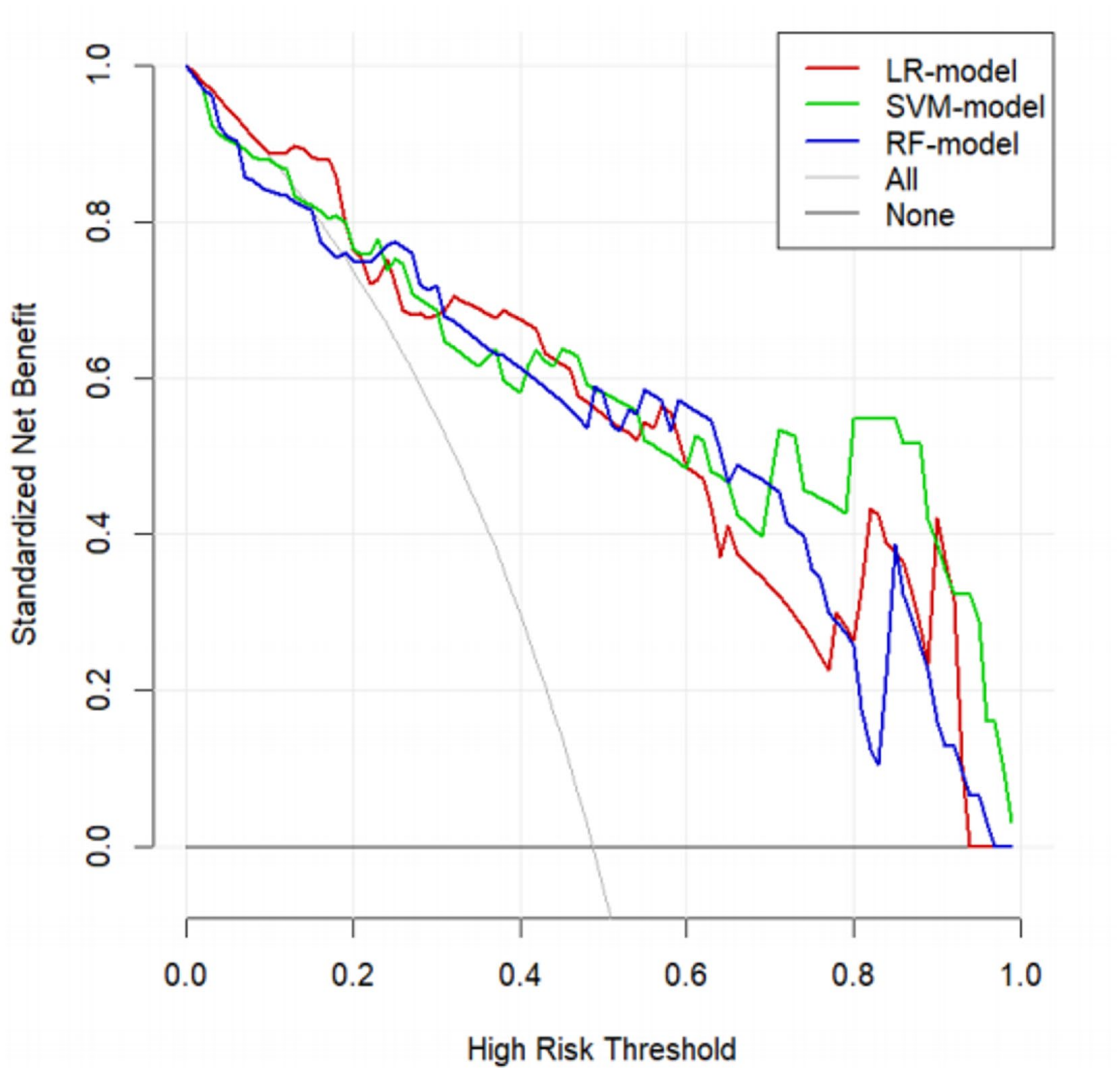
Decision Curve Analysis for Three Different Machine Learning Models. The decision curve analysis (DCA) evaluates the clinical utility of three machine learning models: logistic regression (LR), support vector machine (SVM), and random forest (RF). The standardized net benefit is plotted against the high-risk threshold. The LR-model, SVM-model, and RF-model are represented by red, green, and blue lines, respectively. “All” represents the strategy of treating all patients, while “None” indicates no patients are treated. The RF-model shows a higher net benefit across most risk thresholds, indicating superior clinical utility.

In this study, we evaluated the clinical utility of three machine learning models—Logistic Regression (LR), Support Vector Machine (SVM), and Random Forest (RF)—using Decision Curve Analysis (DCA). The results show that the Random Forest model (RF-model) exhibited the highest net benefit in the lower risk threshold range (0.0 to 0.2). As the risk threshold increased, the Logistic Regression model (LR-model) and the Support Vector Machine model (SVM-model) demonstrated comparable performance; however, the Random Forest model maintained a slight advantage. Notably, in the higher risk threshold range (0.8 to 1.0), the Random Forest model continued to outperform the other two models. Overall, the decision curve analysis indicates that the Random Forest model provides the best clinical utility across a wide range of risk thresholds, suggesting its significant potential as a robust tool for clinical decision-making(Fig. 4).

## Discussion

The main finding of this study lies in the successful development of a random forest model that utilises radiomic features extracted from cone beam computed tomography (CBCT) of the to enable the early detection of carotid atherosclerosis in patients with periodontitis. By analysing 279 observations, each characterised by 206 features, and addressing class imbalance through the application of SMOTE oversampling, we identified a final set of 20 features following a rigorous feature selection process. In five-fold cross-validation, the model demonstrated excellent performance, achieving an AUC of 0.892, sensitivity of 0.957, and specificity of 0.710. Importantly, compared to logistic regression and support vector machine models, the random forest model not only exhibited superior predictive accuracy but also showed broader clinical utility in decision curve analysis, indicating its ability to provide higher net benefit across a range of risk thresholds. This underscores the potential value and applicability of using CBCT-based radiomic features for the early identification of carotid atherosclerosis in patients with periodontitis.

Mounting epidemiological and mechanistic evidence links periodontitis to accelerated carotid atherosclerosis, likely mediated by chronic inflammation, bacterial dissemination, and endothelial dysfunction^6–9,17,18^. In previous clinical work, it has been observed that patients with more severe periodontal disease (PD) face an increased risk of developing coronary artery atherosclerotic heart disease, peripheral arterial disease, and experiencing a first cerebrovascular event^19,20^. Moreover, patients with cardiovascular and cerebrovascular diseases are also at a higher risk of recurrent related events^21^. Measurements of flow-mediated vasodilation, arterial stiffness (e.g., pulse wave velocity), intima-media thickness, and arterial calcification scores have revealed significant endothelial dysfunction in patients with periodontitis^22^. Crucially, no prior research has explored CBCT radiomics as a predictor of carotid pathology—a gap addressed by our study. Recent advances in artificial intelligence and radiomic analysis now enable the decoding of complex, non-intuitive patterns in medical imaging. By applying these techniques to routine dental CBCT scans, we demonstrate that morphometric and textural alterations in periodontal structures reflect systemic vascular risk, establishing a novel, non-invasive diagnostic paradigm. This approach capitalises on existing dental imaging infrastructure, circumventing the need for additional costly or invasive tests.

Recent advances in predicting carotid atherosclerosis have been achieved through the use of advanced computational techniques. One study integrated demographic, clinical, and molecular data with ultrasonographic measurements, employing neural network algorithms and hierarchical clustering to identify four subclinical carotid atherosclerosis endotypes, ranging from mild to severe, within the IMPROVE cohort (*n* = 3340)^23^. This approach not only improved ASCVD risk discrimination but also demonstrated potential applications in precision medicine for ASCVD prevention. Another study utilized deep learning (DL) models to predict cardiovascular disease and coronary artery disease risks based on carotid plaque features^24^. Among 459 participants undergoing coronary angiography, contrast-enhanced ultrasound, and B-mode carotid imaging, parameters such as maximum plaque height, total plaque area, carotid intima-media thickness (cIMT), and intraplaque neovascularization were analysed. Results showed that DL models outperformed traditional machine learning methods, achieving a 21% improvement in AUC and a ~ 17% increase in c-index compared to the Cox proportional hazards model (CPHM). Notably, IPN demonstrated significant predictive power for cardiovascular events (*p* < 0.0001). Additionally, advancements in cardiovascular modelling have enabled the creation of patient-specific three-dimensional carotid artery models^25^. This study combined non-imaging and imaging data with simulated haemodynamic data to develop a prognostic model for carotid stenosis progression, achieving 71% accuracy using a neural network classifier. The novelty of this work lies in its unique problem definition and extensive use of simulated data as input for the predictive model. These studies collectively highlight the potential of integrating advanced machine learning techniques with detailed clinical and imaging data to enhance the prediction and management of carotid atherosclerosis.

In our study, the Random Forest model’s exceptional performance, with a sensitivity of 0.957, highlights its potential as an effective screening tool. The frequent selection of intensity-based features, such as Kurtosis and Skewness, further provides a mechanistic link between oral inflammation and atherosclerosis. This suggests not only the practical utility of the model in clinical settings but also deepens our understanding of the underlying pathways connecting periodontal health to cardiovascular disease. A cross-sectional study conducted in Japan found that both the absence of regular dental check-ups and the presence of periodontitis were significantly associated with atherosclerosis among community-dwelling residents^26^. This suggests that maintaining good oral health may play an important role in reducing the risk of cardiovascular diseases. Further research has highlighted the critical role of inflammatory responses in this association. A case-control study indicated that systemic inflammatory responses triggered by inflammatory cytokines, bacterial pathogens, and altered lipoprotein metabolism in patients with periodontitis may promote the development and progression of atherosclerosis^27^. The study also demonstrated that clinical attachment loss (CAL) and carotid intima-media thickness (IMT) were significantly higher in individuals with periodontitis compared to healthy controls, underscoring the significant role of inflammation in the atherosclerotic process. Additionally, a study conducted among Korean adults further supported this view, showing a close relationship between severe periodontitis and the development of early atherosclerotic vascular disease, particularly among non-smokers^28^. As the severity of periodontitis increased, the adjusted mean carotid intima-media thickness (cIMT) significantly increased, while the ankle-brachial index (ABI) decreased, indicating a dose-dependent negative impact of periodontitis on atherosclerosis. Evidence from studies on specific populations also provided additional insights. For instance, among individuals with heterozygous familial hypercholesterolemia (hFH), severe periodontitis was associated with higher diastolic blood pressure (DBP), suggesting that severe periodontitis may be an important factor contributing to elevated cardiovascular risk in this population^29^.

Taken together, these studies collectively reveal that periodontitis accelerates the progression of atherosclerosis by inducing systemic inflammatory responses, leading to dyslipidemia, elevated blood glucose levels, and changes in other traditional cardiovascular risk factors. Specifically, periodontal pathogens and their by-products may reach distant organs via the bloodstream, triggering chronic low-grade inflammation, which subsequently impairs vascular endothelial function and ultimately promotes the occurrence and development of atherosclerosis.

In terms of discriminative accuracy and clinical utility, as demonstrated by Decision Curve Analysis (DCA), the Random Forest (RF) model(AUC: 0.892) outperformed Logistic Regression (LR) and Support Vector Machine (SVM). This superiority of RF likely stems from its ability to handle non-linear relationships and feature interactions, which may better capture the complex biological interplay between periodontitis and carotid stenosis. Notably, in the third fold, the model maintained high sensitivity (0.957), suggesting its potential as a screening tool for high-risk patients. However, its specificity varied between 0.667 and 0.743 (0.710 in the third fold), indicating some variability and implying that false positives could limit diagnostic precision in low-prevalence populations. Although SVM showed a slightly higher AUC than RF in the third fold (0.897 vs. 0.892), the overall performance of RF still demonstrates a good balance between sensitivity and specificity.

Our feature selection methodology—combining correlation filtering, LASSO regression, and bootstrap stability analysis—enhanced the generalizability of the radiomic signature^30,31^. Nevertheless, the clinical translation of radiomics in periodontitis remains nascent. While previous research has focused on imaging biomarkers for periodontal bone loss^32^, this study extends their application to systemic vascular comorbidity, offering a novel diagnostic paradigm.

The high-frequency selection of Original_firstorder_Kurtosis and Original_firstorder_Skewness suggests that intensity distribution abnormalities in periodontal tissues may serve as early markers of vascular involvement. However, the exact mechanistic basis for these radiomic patterns warrants further investigation, particularly whether they reflect localized inflammation or systemic endothelial dysfunction.

Despite these advances, several limitations must be acknowledged. First, the retrospective, single-center design limits causal inference and introduces potential selection bias. Second, the absence of an external validation cohort constrains the assessment of the model’s generalizability to other populations and imaging protocols. Third, the reliance on SMOTE-augmented data, while mitigating class imbalance, may introduce synthetic patterns that do not fully represent biological variability; future studies with larger, naturally balanced cohorts are needed. Fourth, we only evaluated three interpretable machine learning models (LR, SVM, RF). Although these provided strong performance, future work should explore other architectures such as Artificial Neural Networks (ANN), K-Nearest Neighbors (KNN), or Decision Trees (DT) to potentially achieve higher accuracy or different insights. Fifth, important clinical confounders such as detailed medication use (e.g., statins, anti-inflammatory drugs), periodontal severity indices (e.g., full-mouth plaque/bleeding scores), and carotid plaque characteristics were not integrated into the current model, which may affect predictive precision. Sixth, the radiomic feature stability requires further investigation regarding test-retest reproducibility, inter-observer segmentation variability, and robustness across different CBCT scanners. Finally, while the bootstrap feature selection is rigorous, the biological interpretability of the selected radiomic features and their direct link to the pathophysiology linking periodontitis and atherosclerosis warrant deeper mechanistic investigation.

## Conclusion

This study developed and validated a random forest model based on CBCT radiomic features for the early detection of carotid atherosclerosis in periodontitis patients. The model demonstrated excellent predictive performance (AUC = 0.892, sensitivity = 0.957, specificity = 0.710) and showed high clinical utility in decision curve analysis, indicating its potential value in early detection.

## Data Availability

All data generated or analyzed during this study are included in this published article. The raw data that support the findings of this study are available from the authors without undue reservation. The datasets supporting the conclusions of this article can also be obtained from the corresponding author upon reasonable request.
